# Prevention of Periodontal Pocket Formation after Mandibular Third Molar Extraction Using Dentin Autologous Graft: A Split Mouth Case Report

**DOI:** 10.1155/2020/1762862

**Published:** 2020-08-31

**Authors:** Alberto De Biase, Giulia Mazzucchi, Dario Di Nardo, Marco Lollobrigida, Giorgio Serafini, Luca Testarelli

**Affiliations:** Department of Oral and Maxillo-Facial Sciences, Sapienza University of Rome, Italy

## Abstract

Surgical extraction of the third molar can often result in the development of a periodontal pocket distal to the second molar that could delay the healing, and the socket could be colonized by bacteria and lead to secondary abscesses, or it may cause mobility or hypersensitivity. The aim of this case report is to assess the efficacy of a dentin autograft in the prevention of periodontal dehiscences after the surgical extraction of the third molar, obtained by the immediate grinding of the extracted tooth. A healthy 18-year-old male patient underwent surgery of both impacted mandibular molars: right postextractive socket was filled with grinded dentin; then, the left one was filled with fibrin sponge. The patient was followed up for six months, and clinical and radiographic assessment were performed: measurements of plaque index (PI), bleeding on probing (BOP), gingival index (GI), clinical attachment level (CAL), and probing pocket depth (PPD) were done before surgery and repeated at 90 and 180 days after the extractions. Measurements made at six months after the surgery revealed that the grafted site was characterized by a minor depth of the pocket if compared with the nongrafted site, with no clinical/radiographic signs of complications.

## 1. Introduction

Surgical extraction of the third molar can often result in the development of a periodontal pocket distal to the second molar: this condition could delay the healing and the socket could be colonized by bacteria and lead to secondary abscesses or it may cause mobility, hypersensitivity, or the formation of a periodontal pocket. The formation of a dehiscence can be due to other factors like the adopted surgical technique (extractive procedures and suture), postoperative care of the wound, and occurrence of complications during and after the procedures (iatrogenic errors, infection, retraction, resorption or collapse of the flap, and foreign body reaction) [1–6].

The socket preservation technique is considered an effective procedure to preserve the alveolar bone from the postextractive physiological resorption [7, 8]. However, as like other surgical intervention, it can be affected by the same complications that could lead to a failure of the graft (exposition of the graft, infection, resorption, loss of grafted material, and adverse reaction to the grafted materials) [9]. Different types of grafts can be used for the scope: synthetic grafts, xenografts (from other species), allografts (from human donors), and autografts (from the patient itself). Each type of graft is characterized by different morbidity, risk of diseases transmission, grade of resorption, osteoinductivity, and osteogenicity. However, actually, only the autologous bone graft is characterized by nonimmunogenic and nonpathogenic properties, with osteoinductive, osteoconductive, and osteogenic properties, and it is widely considered the gold standard [10–12].

Socket preservation is usually performed with commercial products (e.g., NanoBone®, Artoss GmbH, Rostock, Germany; Geistlich Bio-Oss®, Geistlich Pharma AG, Wolhusen, Switzerland; and Laddec®, Biohorizons, Birmingham, AL, USA) that could be expensive, and for so, they are preferred to be used only when the positioning of an implant is expected or when aesthetic purposes are requested: autogenous tooth graft is considered to be an osteoconductive material, characterized by a high bone formation activity and biocompatibility [9, 13–15].

Since commercial alloplasts, allografts, and xenografts are considered safe for human purposes and their outcomes are mostly successful, the possibility of retrieving an effective and ready-to-use grafting material directly from a tooth that is going to be extracted could result advantageous for the patient and for the clinician in terms of saving time and reducing costs, with the same outcome of other products. The use of autologous dentin is obviously cheaper than other grafts and it is fully compatible with the patient tissues. Furthermore, it is immediately available and its amount usually corresponds exactly to the amount of graft needed to fill up the postextractive socket [16–18].

The aim of this case report is to assess the efficacy of a dentin autograft in the prevention of periodontal dehiscences after the surgical extraction of the third molar, obtained by the immediate grinding of the extracted tooth.

## 2. Case Presentation

An asymptomatic, generally and periodontally healthy 18-year-old male, nonsmoker and nonalcohol addicted patient needing the extraction of both mandibular third molars was enrolled for the case. A written informed consent was obtained prior to start the procedure. All the procedures were conducted according to the declaration of Helsinki, and the protocol was approved by the Policlinico Umberto I of Rome Ethical Committee (n. 5456/2019).

**Figure 1:**
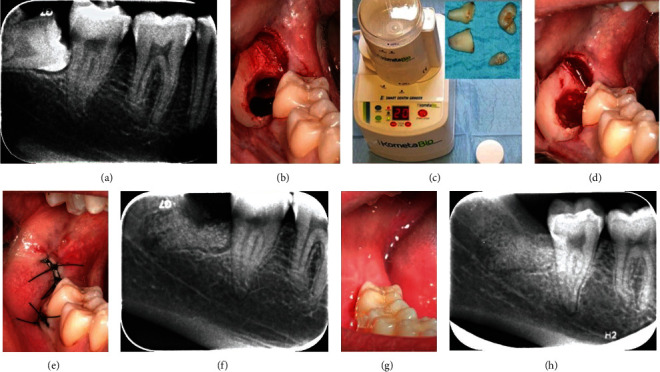
Presurgical periapical radiograph of the right third molar (test site) (a). Fresh post-extractive socket (b). The Smart Dentin Grinder™ (c) used for the test and the fragments of the tooth before being grinded (square). The dentin particulate filling the socket (d). The sutured wound (e). Periapical radiograph of the grafted socket taken after 15 days from the surgery (f). Soft (g) and hard (h) tissues healing at 180 days after surgery.

**Figure 2:**
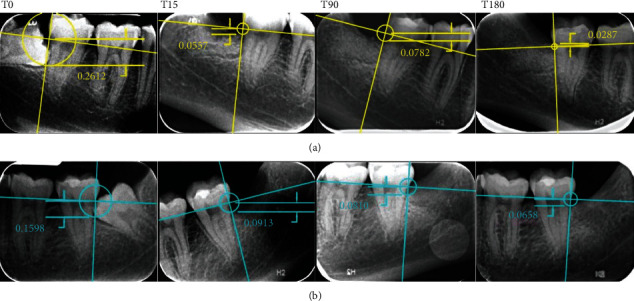
Periapical radiographs of the grafted site (a) and the control site (b) before surgery (T0) and after 15, 90, and 180 days. Since the positioning of the film in a so posterior area resulted difficult, a rigid support held by the patient was adopted instead of the Rinn centrator. Adjustments to tilted images were performed by the same software used for the measurements.

Prior to extractions, periapical radiographs of the interested third molars were taken, and the following periodontal indexes were recorded by a calibrated and blinded operator (G.S), at mesiobuccal, midbuccal, distobuccal, distolingual, midlingual, and mesiolingual sites of the bilateral mandibular second molars: plaque index (PI), bleeding on probing (BOP), gingival index (GI), clinical attachment level (CAL), and probing pocket depth (PPD) were recorded by the use of a PCP-15-UNC probe (Hu-Friedy, Leimen, Germany) (Table 1) [19, 20]. The registration of all those above-mentioned periodontal indexes was considered mandatory due to avoid invalid results caused by the shrinkage of the soft tissues during the healing. Measurements were repeated at 90 and 180 days after surgery by the same operator, and periapical radiographs were taken at every follow-up appointment. Since Rinn centrator was unable to reach a so posterior area, a rigid support for articulation paper was adopted to stabilize the radiographic film. The patient was instructed to keep the rigid support in contact and as much as possible parallel to the occlusal surface, and the radiograph was taken only after the operator's supervision due to standardizing the exam. To keep the irradiation as low as reasonably achievable (ALARA), the authors decided to not repeat the radiographic exam if small imperfections were present (Figures 1 and 2).

A 60 second 0.2% chlorexidine mouth rinse was administered before surgical procedures. Surgical interventions were all performed by the same skilled clinician (M.L). A full-thickness flap [21] was performed by the use of a Bard-Parker 15c scalpel, and the periostium was elevated by the use of a Molt elevator. Odontotomy was performed by the use of a multiblade cutter in tungsten carbide and mobilized by levers of different thickness, taking in considerations common recommendations to minimize the risk of intraoperational accidents [22].

Immediately after the extraction (Figure 1), the right molar's fragments were grinded for 3 seconds (300-1200 *μ*m) by the use of the Smart Dentin Grinder™ (KometaBio, Fort Lee, NJ, USA) and then immersed into a 0.5 M NaOH and 20% ethanol solution for 10 minutes, in order to dissolve all the organic remains from the tubules. The particulate was first dried with a sterile gauze, then rinsed two times (3 minutes each) with phosphate-buffered saline solution to remove all the NaOH and the ethanol [16, 23]. The right postextractive socket (test site) was filled with the grinded dentin; then, the left one (control site) was filled with a fibrin sponge.

Surgical flaps were stabilized with a 4.0 nylon suture (Ethilon, Johnson & Johnson, New Brunswick, NJ, USA), and both wounds were allowed to heal for first intention.

The patient was instructed to properly clean wounds with a soft surgical tooth brush and to not rinse or spit for the following 48 hours, to not practice sport for two weeks, and to avoid smoke or alcohol until suture removal.

Antibiotics were administered for 6 days (1 tablet of amoxicillin 875 mg + clavulanic acid 125 mg every 12 hours), and chlorhexidine digluconate 0.20% rinses were performed twice a day for two weeks.

Analgesic drugs were administered orally after the intervention (nimesulide 100 mg) and prescribed to assume in case of pain every 10 hours.

Sutures were removed after 14 days, and periapical radiographs were taken in order to assess the conditions of the postextractive sockets. At 90 and 180 days, new periapical radiographs were taken, and periodontal parameters were reevaluated for both sites. A period of 180 days of follow-up was chosen since a period of 20-21 weeks is considered sufficient to obtain evidences about the status of the regenerated bone, as showed in many histological randomized controlled trials [24–27].

At the day of surgery, the distal pocket of the second molar measured 4 mm for both sites. After 3 months from the extraction, a reduction of pocket depth at the test site of 1 mm was observed, while the control test still measured 4 mm: no changes to these measurements were recorded after 6 months from the surgery (Table 1).

Differences between the postextractive sockets and changes occurred during the follow-up period were measured by the software AutoCAD 2017 (Autodesk, San Rafael, CA, USA) on periapical radiographs taken at different times and then validated by Invivo software (Anatomage Inc., San Jose, CA, USA) and 3D Endo Software (Dentsply Sirona, Charlotte, NC, USA) by a blinded operator (G.M). Measurements were performed from the CEJ to the bone peak distal to the second molar, on a line perpendicular to the one passing through the mesial and distal projection of the CEJ (Figure 2). Results of the measurements are reported in Table 2.

## 3. Discussion

Dental elements that are adjacent to an impacted or semi-impacted tooth are usually more predisposed to periodontal defects [28]. Obviously, it is not mandatory to perform socket preservation for every extracted third molar; however, it can be useful in those cases, like surgical extractions, where a great amount of bone can be lost, and this could lead to a periodontal defect which could lead to mobility or hypersensitivity of the second molar [29]. The amount of bone that could be retrieved during the osteotomy could be not enough for the dimension of the dehiscence: for so, it would be necessary additional bone or graft material to completely fill the postextractive socket. Other risk factors associated with the development of periodontal pocket distal to the second molar are considered the design of the flap, a preexistent bone defect, the age of the patient, and the distance between the adjacent teeth [30, 31].

The development of a periodontal defect distal to the second molar is not predictable, and it is considered a complication difficult to treat and that will not heal spontaneously. There is consensus that the best way to prevent distal defects is the early extraction of third molars [32–35], but when this condition is not applicable, the risk of bone loss should be always taken in consideration. Richardson and Dodson revealed that after 2 years from the extraction of impacted third molars, the 43.3% of the patients showed a PPD of ~7 mm [29].

Autologous dentin graft was investigated by Kim et al. by scanning electron microscopy, X-ray diffraction analysis, and calcium/phosphate dissolution test: results were compared with human bone and other heterologous, homologous, and allopastic grafting materials, revealing that the autologous dentin graft was the most similar to the human bone for structure and physico-chemical characteristics [36].

A study performed by the use of SEM-energy dispersive X-ray (SEM-EDX) measured the percentages of calcium (23.42 ± 0.34%) and phosphate (9.51 ± 0.11%) in the dentin of human extracted teeth and revealed a similarity with the bone [37]. Calvo-Guirado et al., in a study on beagle dogs, hystologically demonstrated the formation of new well-organized bone, with multiple osteons at 90 days after dentin autograft. Hystomorphometry revealed the presence of higher percentages of mature bone and less percentages of immature bone in the sites grafted with particulated dentin when compared with no-grafted sites at 90 days [23].

Calvo-Guirado et al. suggested the use of sound retained or redundant roots to preserve postextractive alveolar sockets: in an animal model, an effective osteointegration of dental implants was demonstrated histologically, with resorption of the grafted dental tissues and the formation of new vascularized bone [38].

Another advantage of the grinded tooth is that the volume of the particulate results to be 2-3 times greater than the volume of the entire tooth: furthermore, the presence of spaces between the particles and the presence of micro- and macropores could allow the infiltration of blood vessels and osteogenic cells [17].

In literature, it has been reported that human dental tissues contain different growth factors similar to the bone: IGF-II, BMP-2, and TGF-b are present in the dentin; TGF-b, IGF-I, and PDGF-BB are present in cementoblasts. It has been reported that such growth factors and type I collagen can be found also in the periodontal ligament, in association with VEGF and basic fibroblast growth factors. [39, 40] However, the use of NaOH and ethanol for the removal of the organic materials could incapacitate the action of such growth factors, and eventually, stem cells present in the pulpal tissues [17].

**Figure 3:**
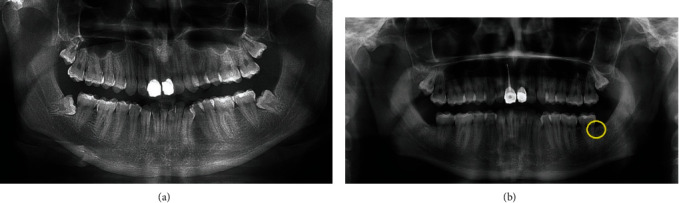
Panoramic radiographs of the patient before surgery (a) and after 6 months: left pocket (yellow circle) still resulted to be deeper than the grafted one (b). No signs of adverse reaction or bone resorption were present at the grafted site.

Results from this case report showed an enhanced reduction of bone loss in the grafted site if compared with the control one: when measuring the distance between the CEJ and the bone peak, the control site depth obtained a reduction of 0.94 mm instead of 2.32 mm of the grafted site. Clinical probing depth resulted to reduce of 1 mm in the grafted site while no changes were recorded in the control site from the day of the surgery. Results can be explained by the presence of grafting material which avoided the apical migration of the soft tissues [14]. For what concerns the control site, it was filled with fibrin sponge, that is, a rapid resorbable material, which is unable to avoid soft tissue invasion of the postextractive socket. Since the patient was informed on the possible complications of the surgical interventions and the possible adverse reaction or failure of the graft (swelling, pain, hemorrhage, dysfagia, alveolitis, displacement, and necessity of reintervention due to removing the graft), he was expected to experience any kind of adverse reactions, but he reported no discomfort even after 6 months from the surgery as like no hypersensitivity or pain at the sites of intervention. No hypersensitivity or mobility of the second molar was evidenced clinically. Furthermore, similarly to other hydroxyapatite grafting materials [41], no adverse reactions or delays in the healing were reported: this can suggest that the dentine autograft is safe, and it can be used effectively as bone substitute (Figure 3). Mazor et al. [42], in a study on grafts performed with autologous tooth particles, obtained with the Smart Dentin Grinder, revealed hystologically, that after three months, vital bone was in direct contact with the tooth particles, without evidence of inflammatory infiltrate. Since results of this case report are in line with other recent studies [16, 17, 23, 36–38, 43, 44], it can be concluded that the dentin autologous graft is a safe mean to preserve alveolar ridge, and its availability and low costs make it an affordable procedure if compared with the more expensive commercial materials. However, further studies are required, and the effectiveness of autologous dentine graft as a substrate for implant placement is still to be assessed.

## Figures and Tables

**Table 1:** Periodontal measurements of the second molar (mm), at different follow-up intervals.

Periodontal indexes		4.7			3.7		
		T1	T90	T180	T1	T90	T180
GI		1	1	0	0	0	1
PI	Buccal	1	0	0	2	1	1
	Lingual	2	2	2	2	2	2
PPD	Mesiobuccal	1	1	1	1	1	1
	Midbuccal	2	1	1	1	1	1
	Distobuccal	**4**	2	2	3	2	2
	Middistal	**4**	**4**	3	3	**4**	**4**
	Distolingual	**4**	3	2	3	**4**	**4**
	Midlingual	3	2	2	2	2	2
	Mesiolingual	3	3	2	3	2	2
BOP	Mesiobuccal	—	—	—	—	—	—
	Midbuccal	—	—	—	—	—	—
	Distobuccal	—	—	—	—	—	—
	Middistal	—	—	—	+	+	+
	Distolingual	+	+	—	—	—	+
	Midlingual	+	+	—	—	—	—
	Mesiolingual	+	+	—	—	—	—
CAL	Mesiobuccal	1	1	1	1	1	1
	Midbuccal	2	1	1	1	1	1
	Distobuccal	**4**	2	2	3	2	2
	Middistal	**4**	**4**	3	3	**4**	**4**
	Distolingual	**4**	3	2	3	**4**	**4**
	Midlingual	3	2	2	2	2	2
	Mesiolingual	3	3	2	3	2	2

**Table 2:** Radiographic measurements from CEJ to bone peak (mm) at different follow-up intervals.

Test site (right)				Control site (left)			
Day 0	Day 15	Day 90	Day 180	Day 0	Day 15	Day 90	Day 180
2.612	0.557	0.782	0.287	1.598	0.913	0.81	0.658
